# Association between Toll-Like Receptor 4 Expression and Neural Stem Cell Proliferation in the Hippocampus Following Traumatic Brain Injury in Mice

**DOI:** 10.3390/ijms150712651

**Published:** 2014-07-17

**Authors:** Yuqin Ye, Hongyu Xu, Xin Zhang, Ze Li, Yanfeng Jia, Xiaosheng He, Jason H. Huang

**Affiliations:** 1 Department of Neurosurgery, Xijing Hospital, Fourth Military Medical University, Xi’an 710032, China; E-Mails: chinayeyuqin@163.com (Y.Y.); xuhy@fmmu.edu.cn (H.X.); heartychang@163.com (X.Z.); lizewyl@163.com (Z.L.); sjwkjyf@126.com (Y.J.); 2 Department of Neurosurgery, Second Affiliated Hospital of Hunan Normal University (PLA 163 Hospital), Changsha 410000, China; 3 Department of Neurosurgery, Baylor Scott & White Health, Temple, TX 76508, USA; E-Mail: JHUANG@sw.org

**Keywords:** toll-like receptor 4 (TLR4), neural stem cells (NSCs), hippocampus, traumatic brain injury (TBI), mice

## Abstract

Whether or how neural stem cells (NSCs) respond to toll-like receptor 4 (TLR4) in an inflammatory environment caused by traumatic brain injury (TBI) has not been understood. In the present study, association between TLR4 expression and NSCs proliferation in the hippocampus was investigated in a mouse model of TBI using controlled cortical impact (CCI). Hippocampal proliferating cells were labeled with the thymidine analog 5-bromo-2-deoxyuridine (BrdU). In order to identify NSCs, the proliferating cells were further co-labeled with BrdU/sex determination region of Y chromosome related high mobility group box gene 2 (SOX2). Morphological observation on the expression of BrdU, SOX2, and TLR4 in the hippocampus was performed by inmmunofluorescence (IF). Relative quantification of TLR4 expression at the protein and mRNA level was performed using Western blotting and real-time polymerase chain reaction (PCR). It was observed that BrdU^+^/SOX2^+^ cells accounted for 95.80% ± 7.91% among BrdU^+^ cells; several BrdU^+^ cells and SOX2^+^ cells in the hippocampus were also TLR4-positive post injury, and that BrdU^+^ cell numbers, together with TLR4 expression at either protein or mRNA level, increased significantly in TBI mice over 1, 3, 7, 14, and 21 days survivals and changed in a similar temporal pattern with a peak at 3 day post-injury. These results indicate that hippocampal proliferating cells (suggestive of NSCs) expressed TLR4, and that there was a potential association between increased expression of TLR4 and the proliferation of NSCs post TBI. It is concluded that hippocampal TLR4 may play a potential role in endogenous neurogenesis after TBI.

## 1. Introduction

In recent years, it has been documented that neurogenesis occurs not only in the immature brain, but also in the adult mammalian brain; and adult brain neurogenesis is a complex process that requires neural stem cells (NSCs) to proliferate, differentiate, migrate and integrate into existing networks [1,2]. NSCs are mainly located in two regions of the adult brain: the subgranular zone (SGZ) of the hippocampal dentate gyrus (DG), and the subventricular zone (SVZ) of lateral ventricles. These cells have a potential capacity of self-renewal, and can give rise to new neurons, oligodendrocytes, and astrocytes. Neurogenesis is often activated in some pathological conditions and plays a crucial role in neural regeneration and repair. Several investigations showed that adult endogenous neurogenesis was modified by inflammatory responses to neurotrauma [3,4,5]. However, it is not yet clear how the proliferation, differentiation, migration, and integration of NSCs involved in the neurogenesis process is influenced by brain inflammation following TBI.

As important innate immune receptors, toll-like receptors (TLRs) are a pattern-recognition receptor family that recognizes pathogen-associated molecular patterns and endogenous ligands, termed as damage-associated molecular patterns, to mediate frontier defense in the central nervous system (CNS) [6]. Toll-like receptor 4 (TLR4) is the first identified mammalian homolog in the TLR family. Accumulating evidence supports that TLR4 is expressed in diverse cell types of the brain including microglia, astrocytes, oligodendrocytes, and neurons [7], and plays an important role in the identification of an inflammatory environment in CNS-related disorders, such as cerebral hemorrhage, ischemia and trauma [8]. Recent studies show that TLR4 is also expressed on the surface of NSCs and is involved in the proliferation and differentiation of NSCs [9,10,11]. However, the potential role of TLR4 in neurogenesis is not quite clear; whether or how endogenous NSCs respond to hippocampal TLR4 in an inflammatory environment caused by TBI has not been reported. The possibility is very enticing for neurogenesis and brain repair, post-TBI. This study is aimed at investigating TLR4 expression and NSC proliferation in the hippocampus in a mouse model of TBI, and exploring the potential association between hippocampal TLR4 and endogenous neurogenesis (likely by NSCs) following TBI.

## 2. Results

### 2.1. Neurological Deficits and Morphological Abnormality

**Figure 1:**
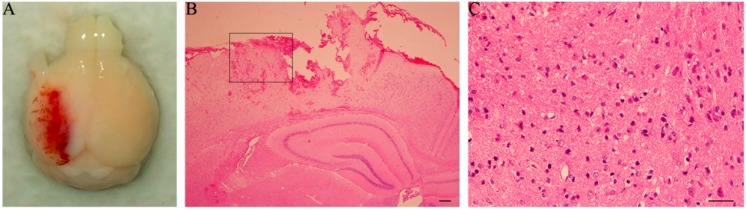
Traumatic brain injury (TBI)-mouse brain and hematoxylin and eosin staining (H&E)-stained coronal section containing hippocampus. (**A**) controlled cortical impact (CCI) produced extensive contusions; (**B**) Diffuse hemorrhages are seen around the contusion focus in the cortex and adjacent to the hippocampus (1 day post trauma); (**C**) The rectangle area in (**B**) was magnified. Massive shrunken neurons were seen with nuclei pyknosis and prominently-stained cytolymph by eosin. Scale bar: 50 μm.

Following controlled cortical impact (CCI), all mice presented with arched back, erect hair, unconsciousness and slow respiration which disappeared 1 h post-injury. They were observed to have paralysis of the right limbs. Brain sections containing the lesion on the cortex 1 day post-trauma were stained with hematoxylin and eosin staining (H&E). Regional cortex contusion and hemorrhages were seen in the damaged brain (Figure 1A), accompanied by massive shrunken neurons with nuclei pyknosis and prominently stained (with eosin) cytolymph (Figure 1B,C).

### 2.2. Neural Stem Cell (NSC) Proliferation

**Figure 2:**
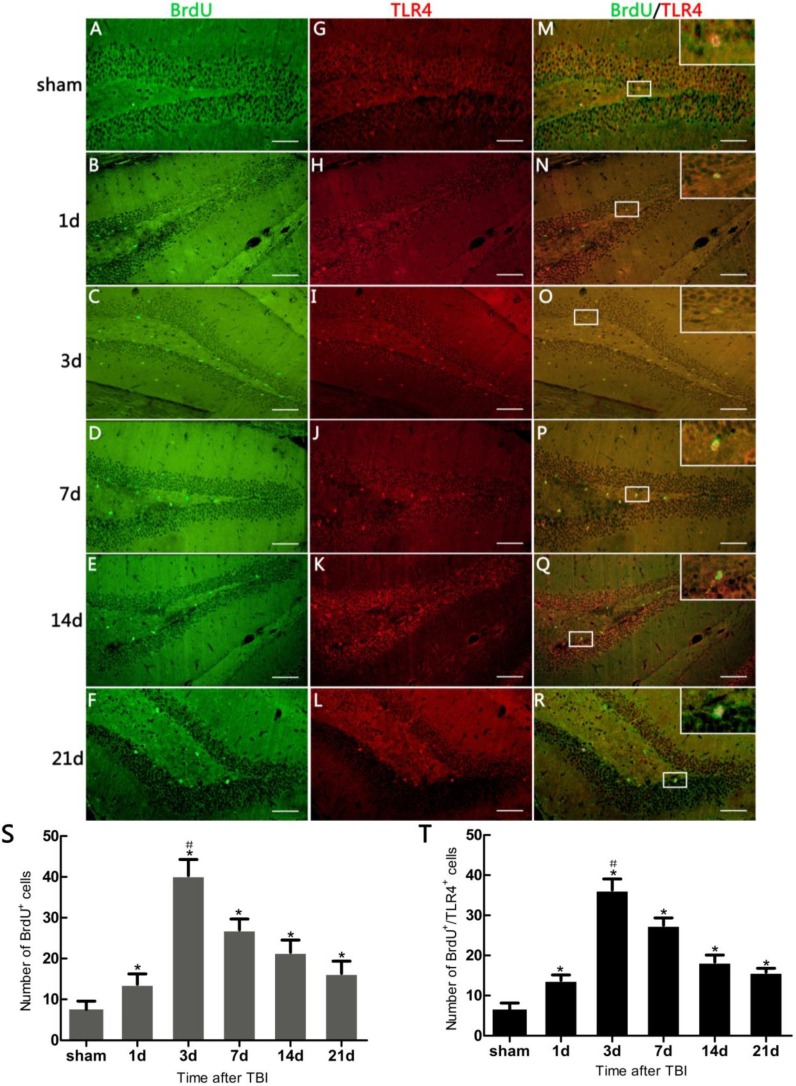
Immunofluorescence (IF) microphotographs in dentate gyrus (DG) of the hippocampus in sham- and TBI-mice with various survivals post-injury. (**A**–**F**) 5-bromo-2-deoxyuridine (BrdU) (green) indicating neural stem cells (NSCs) in DG; (**G**–**L**) Toll-like Receptor 4 (TLR4) (red) indicating TLR4 expression in DG; (**M**–**R**) Merged IF of BrdU/TLR4 indicating TLR4 expression on the proliferating cells (suggestive of NSC proliferation), the rectangle area, taken from a magnified optic field, showing BrdU/TLR4 expressed in/on a newly-generated cell; (**S**)The number of BrdU^+^ cells revealing the degree to which putative NSCs have proliferated in DG varies over different survivals post-injury. TBI-mice has significantly more BrdU^+^ cells than the sham (* *p* < 0.05), and shows an obvious difference in the number of these cells between all post-traumatic time-points (^#^ *p* < 0.05); (**T**) The number of BrdU^+^/TLR4^+^ cells revealing the degree of TLR4 expression in the proliferating cells in DG varies over different survivals post-injury. TBI-mice has significantly more BrdU^+^/TLR4^+^ cells than the sham (* *p* < 0.05), and shows an obvious difference in the number of these cells between all posttraumatic time-points (^#^ *p* < 0.05). Scale bar: 50 μm. Data are presented as Mean ± SD, *n* = 6. SD: standard deviation, d: day.

**Figure 3:**
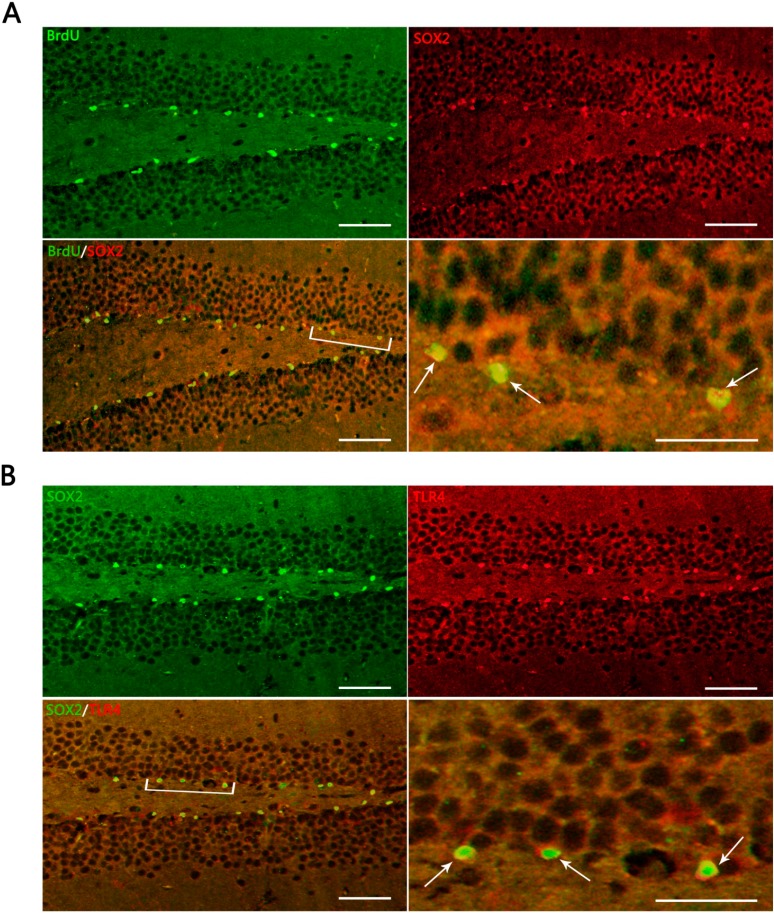
Representative IF microphotographs in the DG of hippocampus in sham- and TBI-mice with various post-injury survival periods. (**A**) BrdU (green), indicating proliferating cells in DG, SOX2 (red), indicating NSCs in DG; merged IF of BrdU/SOX2 indicates that the proliferating cells are presumed, morphologically, to be NSCs (the percentage of putative NSCs in proliferating cells was 95.80% ± 7.91%); the arrows indicate BrdU^+^/SOX2^+^ cells in DG; (**B**) SOX2 (green) indicating NSCs in DG; TRL4 (red), indicating TLR4 expression in DG; merged IF of SOX2/TLR4, indicates TLR4 expression on NSCs, the arrows indicating SOX2^+^/TLR4^+^ cells in DG. The bracketed area is magnified respectively as the right lower part in (**A**) and (**B**). Scale bar: 50 μm; Data are presented as Mean ± SD, *n* = 6.

The number of 5-bromo-2-deoxyuridine (BrdU) positive cells was significantly increased in TBI mice, compared with the sham (*p* < 0.05). BrdU^+^ cells increased at 1 day post-trauma, reached the peak at 3 day, and then reduced gradually (Figure 2S). BrdU/sex determination region of y chromosome related high mobility group box gene 2 (SOX2) co-labeling immunofluorescence (IF) was performed to investigate proliferating cells including NSCs in DG. The proliferating cells were seen with fine green fluor (BrdU^+^ cells), NSCs were observed with fine red fluor (SOX2^+^ cells), the BrdU^+^/SOX2^+^ cells accounted for 95.80% ± 7.91% among BrdU^+^ cells (Figure 3A). This percentage is consistent with an increase of proliferating NSCs in DG.

### 2.3. Expression of Hippocampal Toll-Like Receptors 4 (TLR4) on Inmmunofluorescence (IF) Morphology, Protein, and mRNA Levels

**Figure 4:**
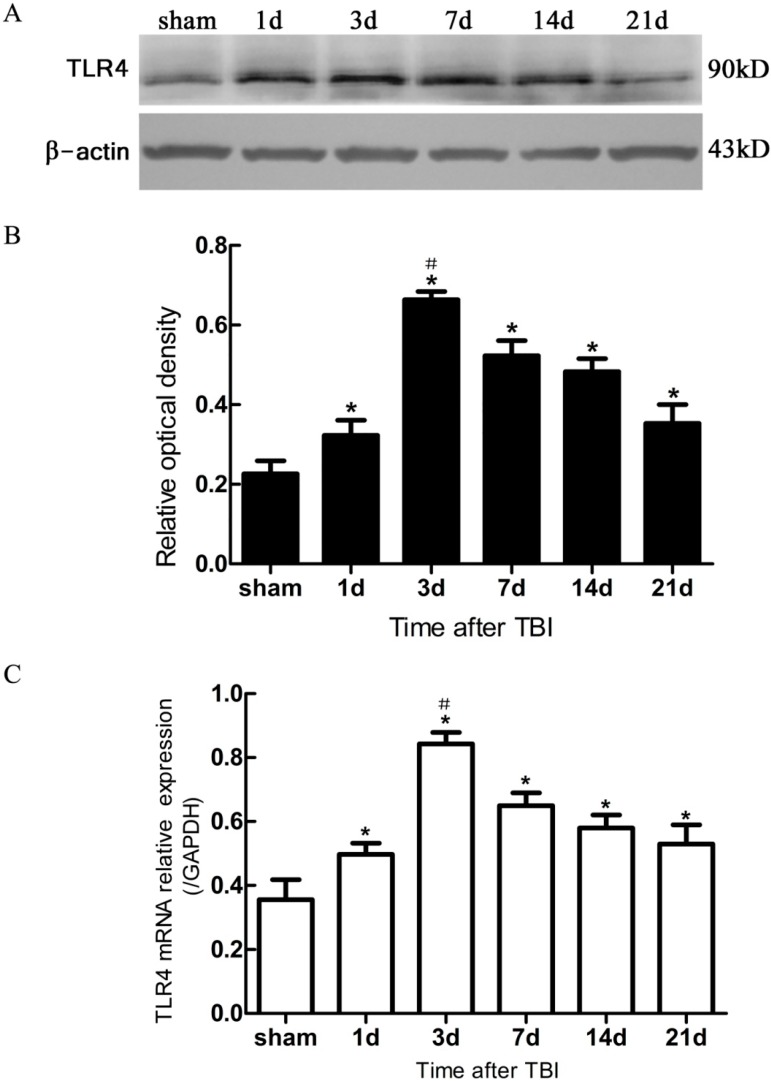
Expression of hippocampal TLR4 protein and mRNA in sham- and TBI-mice, by Western blotting and real-time polymerase chain reaction (PCR). (**A**) Western blotting: electrophoresis bands of TLR4 protein with β-actin as loading control; (**B**) Western blotting: optical density value of TLR4 protein electrophoresis; (**C**) real-time PCR: TLR4 mRNA relative expression, GAPDH was used as endogenous reference gene. TBI-mice had a significantly higher expression of TLR4 in either protein or mRNA than the sham (* *p* < 0.05), and showed an obvious difference in the level of TLR4 expression between all post traumatic time-points (^#^ *p* < 0.05). Data are presented as Mean ± SD, *n* = 6. d: day.

TLR4^+^ cells in IF were observed with fine red fluor on cytomembrane in DG (Figure 2G–L and Figure 3B). SOX2/TLR4 co-labeling IF revealed that TLR4 was expressed on the NSCs (Figure 3B). BrdU/TLR4 co-labeling IF indicated that TLR4 was expressed on the proliferating cells (presumed to be NSCs) in DG (Figure 2M–R). Quantitative assessment showed that the population of BrdU^+^/TLR4^+^ cells increased at 1 day, maximized at 3 day, and then decreased to 21 day post-trauma (Figure 2T). Expression of hippocampal TLR4 protein was up-regulated at 1 day post-trauma and reached the peak at 3 day, then descended gradually over the post-traumatic period at 7, 14 and 21 day, and maintained at a higher level than the sham (*p* < 0.05) (Figure 4A,B). TLR4 mRNA expression also increased at 1 day post-trauma and rose to the peak at 3 day, and fell gradually over the rest of the survival periods to a level still higher than the sham (*p* < 0.05) (Figure 4C).

## 3. Discussion

Neurogenesis after TBI is a consecutive process that includes proliferation and differentiation of NSCs. The main focus of the present study was possible NSCs proliferation, not differentiation. The mice we used were treated with an injection of BrdU, a marker that is a commonly-used method to label the newly-generated, or so-called, proliferating cells in DG [12]. SOX2 is another specific marker of NSCs, and persistently expressed in the nuclear of NSCs and plays a universal role in maintaining NSCs fate in the central nervous system [13]. SOX2 is qualified to characterize NSCs among the proliferating cells in DG. SOX2 expression is relatively stable in NSCs proliferation; however, it is likely to vary in NSCs differentiation into neurons, microglia and astrocytes [13,14]. The results showed that BrdU^+^/SOX2^+^ cells accounted for 95.80% ± 7.91% in BrdU^+^ cells, suggesting that a large majority of the proliferating cells in DG were co-labeled with the two markers (BrdU and SOX2). The localization of these proliferating cells was highly suggestive for NSCs, although a portion could be constituted by other cells such as microglia.

In the present study, it was observed that the expression of both the TLR4 protein and the mRNA in the hippocampus rose at 1 day, peaked as early as 3 day, and gradually reduced over 7, 14, and 21 day survival to a lower value but was still higher than the sham. Simultaneously, the number of BrdU^+^ cells and BrdU^+^/TLR4^+^ cells increased and changed across these survival time-points in a similar way to the above. More importantly, TLR4 was found to be expressed on the putative NSCs *in vivo* by SOX2/TLR4 co-labeling IF.

The parallel temporal pattern in the post-traumatic increase of TLR4 expression and BrdU^+^ cells suggests a possible correlation between the two. It has been documented that NSC proliferation, indentified by increased BrdU^+^ cells, often occurred as a secondary event in TBI [3,12]. In addition, our results suggested that this putative hippocampal NSC proliferation was accompanied by up-regulation of hippocampal TLR4 post-injury. This evidence implies that TLR4 might play a potential role in NSC proliferation following TBI.

Recent studies have shown that endogenous neurogenesis in the hippocampus occurs continuously throughout life [1,2,15]. It is universally acknowledged that neurogenesis is easily triggered in brains that are subject to injuries such as trauma, stroke and aspiration lesions [3]. The injuries initiated, activated, and enhanced the ability of NSCs to generate neurons in the hippocampus. These new neuronal cells are capable of integrating into the existing neuronal networks and contributing to the repair of the hippocampus [15]. In this study of a mouse model of TBI by CCI, the observation of H&E staining in the contused cortex revealed many shrunken neurons with nuclei pyknosis and prominently-stained cytolymph by eosin. These damages coexisted with evidence of possible enhanced neurogenesis (as indicated by proliferation of BrdU-labeled cells) in the hippocampus, with a maximal level present at 3 day post-trauma, but was maintained at a higher than normal level at 21 day. Our findings were consistent with the previous reports regarding adult endogenous neurogenesis following TBI [15].

There has been a growing body of evidence that active neurogenesis in the hippocampus is not only critical for learning and memory, it also improves the other outcomes of TBI [5,16]. However, the endogenous neurogenesis of NSCs is not sufficient to repair the injury caused by TBI. Therefore, strategies to effectively activate the endogenous NSCs to generate more functional neurons are required for tissue reparation and functional restoration post TBI.

Recent investigations focused on evidence revealing TLR4 expression on NSCs and the role of TLR4 in the proliferation and differentiation of NSCs *in vitro*. Roll *et al.* demonstrated that TLR4 was expressed on NSCs from the hippocampus of adult mice [9]. In a study by Covacu *et al.* [11], cultures of NSCs isolated from the SVZ of adult rat brain were exposed to different inflammatory cytokines such as IFN-γ and TNF-α. It was observed that TLR4 was expressed on NSCs with its mRNA level up-regulated in response to IFN-γ, but not TNF-α. Moreover, TLR4 agonists stimulated NSCs to produce TNF-α in the cultures, and had no promoting effect on the proliferation and differentiation of NSCs *in vitro* [11]. On the contrary, relative to the NSCs isolated from wild-type mice, the NSCs in TLR4-deficient mice increased their proliferation and differentiation, but failed to differentiate into mature neurons. It suggested that TLR4 played an important role in both proliferation and differentiation of NSCs *in vitro* [9,10]. Some authors reported that when newly-generated neurons were co-cultured with microglial cells, TLR4 activation led to neuronal death [17], and others held that TLR4 activation had no influence on neuronal survival [18]. These conflicting views might be a result of differences between animal species, experimental models and specific developmental transcriptional stages of NSCs.

TLR4 are also expressed on other types of cells besides NSCs in brain, the TLR4-expressing cells contribute to the process of inflammation and neurogenesis in the neurogenetic niches of brain [5,10,19]. However, the growth conditions for NSC cultures *in vitro* are quite different from those for the neurogenetic niche *in vivo.* There are few studies investigating TLR4 expression in the hippocampus and its potential role in neurogenesis *in vivo* following TBI. In the present study with a mouse model of TBI, we found up-regulated expression of TLR4 at morphological, protein, and mRNA levels in the hippocampus from 1 to 21 day post-TBI *in vivo*. These results are consistent with a previous study reporting an increased expression of Myd88 and NF-κB in the lesioned cortex following TBI with a peak at 3 day post-trauma [20]. Additionally, morphological evidence of TLR4 expression on hippocampal NSCs, and a potential association between TLR4 expression and NSC proliferation in this area were obviously seen in the present study and suggested that TLR4 might play an important role in NSC proliferation after TBI.

Our study investigated the potential association between TLR4 expression and putative NSC proliferation in the hippocampus *in vivo*. It is difficult to differentiate TLR4 expression in protein and mRNA resulting from NSCs and from other cells such as microglia and mature neurons in hippocampus post-injury. This is partly due to the limitations in experimental design *in vivo.* Hence, further studies need to be performed to illustrate precisely the role of TLR4 in pure NSCs in the hippocampus in adult endogenous neurogenesis following TBI.

## 4. Materials and Methods

### 4.1. Animals and Groupings

A total of 108 healthy adult male C57BL/6 mice, weighing 25 ± 5 g, were provided by the Center of Experimental Animals of the Fourth Military Medical University (Xi’an, China). They were maintained in an environment with constant temperature and humidity under a 12 h light-dark cycle and were allowed free access to food and water. Measures were taken to minimize animal pain or discomfort. Experimental procedures were performed in accordance with the Guideline of National Experimental Animals, approved by the Ministry of Science and Technology of China (023915137, 09 January 2001).

An independent group design was adopted. Mice were divided randomly into sham (*n* = 18) and TBI (*n* = 90) groups. The latter was divided equally into 5 subgroups (*n* = 18 each) which were respectively sacrificed on posttraumatic 1, 3, 7, 14, and 21 day. Mouse brains were prepared for IF, Western blotting, and real-time PCR. The sham group was divided into 3 subgroups (*n* = 6 each), which served as a control respectively for each of the above-mentioned preparations.

### 4.2. Traumatic Brain Injury (TBI) Model Establishment

A CCI device (Hatteras Instruments, Cary, NC, USA) was used to produce the mouse TBI as described by Logan *et al.* [21]. The mice were anesthetized intraperitoneally with sodium pentobarbital (60 mg/kg) and then mounted in a stereotaxic frame (Kopf Instruments, Tujunga, CA, USA) of the injury device, secured by ear pins and incisor bar with the head held in a horizontal plane. A midline incision on the scalp was made with sterile procedures, and craniectomy in 4 mm diameter was performed midway between lambda and bregma sutures and midway between the midline and left temporalis muscle. The bone flap was carefully removed without disruption of the underlying dura. A single impact with the end of a piston rod that had a 3-mm diameter contact surface was given perpendicular to the surface of the dura. The downward impact was controlled by the following parameters: 1.0 mm for vertical dura shift, 100 ms for contact time, and 3 m/s for piston velocity. The sham mice were subjected only to crainiectomy without dura impact. The scalp was sutured after injury- or sham-operation. Core body temperature of the animal was maintained at 36–37 °C using a heating pad during surgical procedures and recovery phase.

### 4.3. Administration of 5-Bromo-2-deoxyuridine (BrdU)

The thymidine analog BrdU (Sigma-Aldrich, B9285, St. Louis, MO, USA) was dissolved in sterile saline to a concentration of 10 mg/mL. TBI- and sham-mice were sacrificed immediately after they had been injected intraperitoneally with the BrdU solution (0.01 mL/g) three times, 8 h apart, for labeling the NSCs in hippocampus in order to assess the NSC proliferation.

### 4.4. Tissue Preparation

At scheduled time-points, TBI- and sham-mice were anesthetized intraperitoneally with sodium pentobarbital (60 mg/kg) and perfused intracardially with 20 mL of 0.9% saline through the left ventricle, followed by 4% paraformaldehyde in 0.1 M phosphate buffer saline (PBS) for 60 min. The mouse brain was removed and immersed in 4% paraformaldehyde in PBS (pH = 7.4) at 4 °C overnight, then dehydrated by alcohol and embedded in paraffin. Using a microtome (Leica, Nussloch, Germany), the brain was cut into 5 μm-thick sections coronally through the hippocampus containing the entire DG (from bregma −1.43 to −3.64 mm) and dried at 92 °C overnight. Ten coronal sections (120 μm apart) from each animal were processed for BrdU/TLR4 co-labeling IF. In order to identify the NSCs in the proliferating cells and confirm the TLR4 expression on NSCs post TBI, ten extra brain sections (120 μm apart) through the same plane as above were taken from the six mice in the group with 3 day survival, half of which were processed for IF with either BrdU/SOX2 or SOX2/TLR4 co-labeling.

Severity of TBI was assessed by observation of general behaviors (such as postures, mobility, *etc.*) and post-mortem inspection of the brain damage. Two extra sections, through the same plane as above, were cut from TBI-mice with 1 day survival and treated by H&E staining.

### 4.5. IF

Sections were deparaffinized by alcohol and dimethylbenzene, then incubated in the citric acid antigen retrieval buffer (pH = 6.0) at 95 °C for 15 min to denature the DNA. Non-specific signals were blocked by incubating the sections in PBS with 1% donkey serum albumin and 0.3% Triton X-100 at room temperature for 30 min. For visualization of BrdU, SOX2 and TLR4, the sections were incubated in relevant primary antibody as follows: sheep anti-BrdU antibody (1:200, GeneTex, GTX21893, Irvine, CA, USA) , goat anti-mouse SOX2 antibody (1:400, Santa Cruz, sc-17320, Dallas, TX, USA), and rabbit anti-mouse TLR4 antibody (1:100, ThermoFisher, Scientific, PA5-23124, Rockford, IL, USA) in PBS overnight at 4 °C. Sections were washed 3 times in PBS, then incubated in relevant secondary antibody as follows: Alexa fluor 488-labeled donkey anti-sheep IgG antibody (1:1500, Molecular Probes, U A-11015, Eugene, OR, USA), Alexa fluor 647-labeled donkey anti-goat IgG antibody (1:2000, Molecular Probes, A-21447, Eugene, OR, USA), Alexa fluor 488-labeled donkey anti-goat IgG antibody (1:1000, Molecular Probes, A-11055, Eugene, OR, USA), and Alexa fluor 594-labeled donkey anti-rabbit IgG antibody (1:1500, Molecular Probes, A-11016, Eugene, OR, USA) in PBS for 1 h at room temperature. Finally, the sections were washed 3 times in PBS, mounted with an anti-fade mounting medium containing 1, 4-Diazobicyclo (Electron Microscopy Sciences, CAT17895-01, Hatfield, PA, USA) and cover-slipped. The immunolabeling specificity of the primary antibody was verified by negative controls. All the procedures were performed in a manner that minimized light exposure to the tissue.

### 4.6. Microscopy and Quantification

Brain sections were analyzed by a fluorescence microscopy using an inverted microscopy system (Zeiss, Axiovert 200M, Gottingen, Germany) that was interfaced with a Zeiss Axio Cam MRc5 digital camera controlled by a computer. Images were captured with software (Zeiss, Axio Vision, v4.0, Gottingen, Germany), assembled and labeled in Photoshop 7.0 (Adobe Systems, San Jose, CA, USA).

Proliferating cells in the hippocampus was assessed by counting the BrdU^+^ cells in the DG ipsilateral to the damaged cortex, the anatomic boundary of DG was identified as previously described [22]. The BrdU/TLR4 co-labeling IF sections were observed to evaluate the number of BrdU^+^, TLR4^+^, and BrdU^+^/TLR4^+^ cells. Various positive cells in 5 consecutive visual fields (24.41 µm^2^ each, magnified by 400×) in the DG at each section were counted under fluorescence microscopy. The average across these 5 visual fields was considered as the number of positive cells for each section; and, this value was averaged across the 10 sections and was taken as the final number of positive cells for each brain sample. The data were expressed as Mean ± SD.

With an aim at demonstrating the proliferation of cells in DG post-TBI, cells that were co-labeled with BrdU/SOX2 (two specific markers of NSCs), were quantified using the same procedures as described above. The percentage of BrdU^+^/SOX2^+^ cells among BrdU^+^ cells was calculated. SOX2^+^/TLR4^+^ co-labeling IF sections were analysed for evidence of TLR4 expression on putative NSCs.

### 4.7. Western Blotting

At scheduled time points, mice were anesthetized and sacrificed with their brains removed immediately in the same way as described above in tissue preparation. Hippocampal tissues were dissected from the brain on ice, homogenized and digested in a homogenizer with a lysis buffer containing 1% NP-40, 150 mM NaCl, 50 mM Tris (pH 7.4), 1% Triton X-100, 0.5 mM EDTA, 1 mg/mL aprotinin, 10 mg/mL leupeptin, 1% deoxycholate, and 1 mM phenylmethylsulfonyl fluoride. The lysates were incubated on ice for 15 min and then centrifuged at 12,000 rpm for 30 min at 4 °C. The supernatant was analyzed for protein concentration by bicinchoninic acid Protein Assay kit (Beyotime, P0011, Shanghai, China), sodium dodecyl sulfate (SDS) sample loading buffer was then added, and the sample boiled at 100 °C for 5 min. Samples containing 40 μg protein were separated by 10% sodium dodecyl sulfate-polyacrylamide gel electrophoresis (SDS-PAGE) and transferred from SDS-PAGE to nitrocellulose membranes. The membranes were blocked with Tris-buffered saline solution with 0.1% Tween-20 (TBST) containing 5% low-fat milk, and then incubated with rabbit anti-mouse TLR4 antibody (1:200, Thermo Fisher Scientific, PA5-23124, Rockford, IL, USA) and rabbit anti-β-actin antibody (1:2000, Cwbiotech, CW0097, Beijing, China) overnight at 4 °C. The membranes were washed 3 times in TBST and then incubated with HRP (horse radish peroxidase)-conjugated goat anti-rabbit IgG antibody (1:20,000, Cell Signaling Technology, 7074, Boston, MA, USA) for 1 h at room temperature. The protein bands were visualized in Western Bright ECL solution (Advansta, K-12045-D10, Menlo Park, CA, USA) and analyzed using Gel-Pro Analyzer 6.0 software (Media Cybernetics, Rockville, MD, USA). The ratio of TLR4 to β-actin (control) in gray scale (OD value) was considered as the level of TLR4 protein expression.

### 4.8. Real-Time PCR

At scheduled time-points, mice were sacrificed and hippocampal tissues were isolated from the brain under anesthesia in the same way as mentioned above. Total RNA was extracted using the MiniBEST Universal RNA Extraction Kit (TaKaRa, 9108, Dalian, Japan) according to the manufacturer’s instructions. The concentration and purity of the total RNA was detected by UV spectrophotometry at 260 and 280 nm, which qualified for reverse transcription and amplification. The cDNA was synthesized using PrimeScript™ one step RT-PCR Kit Ver.2 (TaKaRa, RR036A, Dalian, China). For real-time PCR reaction, the SYBR Green RCR kit (TaKaRa, RR820A, Dalian, China) was used in CFX96 real time-PCR detection system (Bio-Rad, Hercules, CA, USA) according to the manufacturer’s instructions. The PCR condition was pre-denaturation at 95 °C for 30 s, followed by 40 cycles of annealing reaction at 95 °C for 5 s, and extension 60 °C for 30 s. GAPDH was used as endogenous reference gene for normalizing the quantities of TLR4 gene expression.

The sequences of the primers used in this study were as follows: TLR4 forward: 5'-CATGGATCAGAAACTCAGCAAAGTC-3'; TLR4 reverse: 5'-CATGCCATGCCTTGTCTTCA-3'; GAPDH forward: 5'-TGTGTCCGTCGTGGATCTGA-3'; GAPDH reverse: 5'-TTGCTGTTGAAGTCGCAGGAG-3'.

### 4.9. Statistical Analysis

All data were presented as Mean ± SD and analyzed using Graphpad Prism software (v.6.01, Graphpad software, San Diego, CA, USA). Differences between groups were assessed by one-way analysis of variance (ANOVA), followed by the least significant difference (LSD) *post hoc* test. The level of significance was set at *p* < 0.05.

## 5. Conclusions

The present study used a mouse model of TBI via CCI and indicated that many BrdU^+^ cells in the hippocampus were also TLR4-positive post-injury, and increased TLR4 expression was followed by an obvious cell proliferation, suggestive of formation of new NSCs. TLR4 expression and, this putative NSC proliferation varied in a similar temporal pattern over post-traumatic survivals. This suggests that there is a likely association between TLR4 up-regulation and NSC proliferation in the hippocampus, and that TLR4 might play a potential role in endogenous neurogenesis in the hippocampus after TBI. Our results revealed an obvious NSC proliferation in the hippocampus, but did not indicate into what cell type the NSCs would be differentiated. Because there is a mixture of newly-generated neurons, microglia, and other types of cells through the process of NSC differentiation, the use of specific markers to identify what cell types these NSCs will differentiate into following TBI remains to be determined in future investigations.
